# Research progress on recombinant NDV in cancer therapy

**DOI:** 10.3389/fimmu.2025.1735440

**Published:** 2025-12-17

**Authors:** Jiating Sun, Jia Wang, Min Xiao, Liming Chen, Yi Guan

**Affiliations:** 1 Guangdong-Hong Kong Joint Laboratory of Emerging Infectious Diseases, Joint Institute of Virology (STU/HKU), Shantou University, Shantou, Guangdong, China; 2 Department of Oncology, First Affiliated Hospital of Shantou University Medical College, Shantou, Guangdong, China; 3 State Key Laboratory of Emerging Infectious Diseases, School of Public Health, The University of Hong Kong, Hong Kong, Hong Kong SAR, China; 4 Shanghai Institute of Virology, Shanghai, China

**Keywords:** cancer treatment, gene editing, immunotherapy, Newcastle disease virus, oncolytic virotherapy

## Abstract

Newcastle disease virus (NDV) has emerged as a promising oncolytic agent in cancer therapy. NDV not only directly lyses tumor cells but also activates the host’s innate and adaptive immune responses, demonstrating potent antitumor activity. However, the efficacy of wild-type NDV is often limited and inconsistent. Advances in genetic engineering have led to the development of a new generation of highly effective and safe recombinant Newcastle disease viruses (rNDVs) by deleting non-essential viral genes or incorporating exogenous functional genes. These genetically engineered NDVs further enhance antitumor activity and optimize the tumor microenvironment by increasing pro-inflammatory cytokine secretion and inducing systemic antitumor immunity. In this review, we summarize the current status of rNDVs, modification strategies, antitumor mechanisms, clinical applications, and combination therapies involving rNDVs. We also discuss the current challenges in utilizing NDV for cancer therapy, including determining the most effective delivery routes, developing strategies to evade neutralizing antibodies, overcoming tumor heterogeneity, and identifying relevant biomarkers.

## 1 Introduction

Cancers represent one of the most significant public health challenges worldwide. Immunotherapy has demonstrated promising potential in cancer treatment; however, it still faces challenges, including limited response rates, drug resistance, and immune-related adverse events (irAEs) (1, 2). The immunosuppressive tumor microenvironment (TME) constitutes a major barrier to the therapeutic efficacy of immunotherapy. Therefore, developing novel therapeutic modalities that can specifically target tumors while simultaneously reversing the immunosuppressive microenvironment has become an urgent priority and a frontier direction in cancer research.

Oncolytic virus (OV) therapy, a highly promising platform for tumor treatment, has attracted significant research interest. OVs have dual mechanisms of action: first, they selectively replicate within tumor cells and directly lyse them, producing the “oncolytic” effect (3); second, and more importantly, the viral replication and tumor cell lysis stimulate robust systemic anti-tumor immune responses in the host (4–8).

Newcastle disease virus (NDV) is an oncolytic virus known for its high safety profile. It primarily infects avian species and exhibits extremely low pathogenicity in humans, providing a strong basis for its clinical safety. In most tumor cells, the antiviral interferon signaling pathway is suppressed, allowing NDV to evade immune clearance and replicate extensively within these cells (9–12). In addition to directly lysing tumor cells, NDV activates the host’s innate and adaptive immune responses, demonstrating significant antitumor activity (13).

Several wild-type NDV strains, such as PV701 and 73-T, have entered clinical trials, demonstrating favorable safety profiles and antitumor efficacy. Although wild-type NDV inherently targets tumors and exhibits immunogenicity, its oncolytic effectiveness remains limited. This limitation primarily results from factors such as neutralization by pre-existing antibodies, insufficient replication efficiency, and impaired immune responses. The development of reverse genetics systems allows researchers to edit the NDV genome precisely, generating rNDV strains with significantly enhanced oncolytic efficacy. Current strategies primarily focus on modifying viral F or HN proteins to improve targeting, optimizing F protein cleavage sites to increase replication capacity within tumor cells, and utilizing NDV as a vector to express various exogenous genes. These recombinant viruses not only retain NDV’s inherent advantages but also exert localized effects within the TME through the expression of functional proteins, synergistically amplifying antitumor immune responses to ultimately achieve a “1 + 1>2” therapeutic effect.

Therefore, this article aims to provide a systematic review of research progress on rNDV in tumor therapy. By examining its modification strategies, mechanisms of action, preclinical and clinical research outcomes, and analyzing current challenges and future directions, it seeks to offer guidance for further research and clinical translation in this field.

## 2 Modification strategies for recombinant NDV

**Figure 1:**
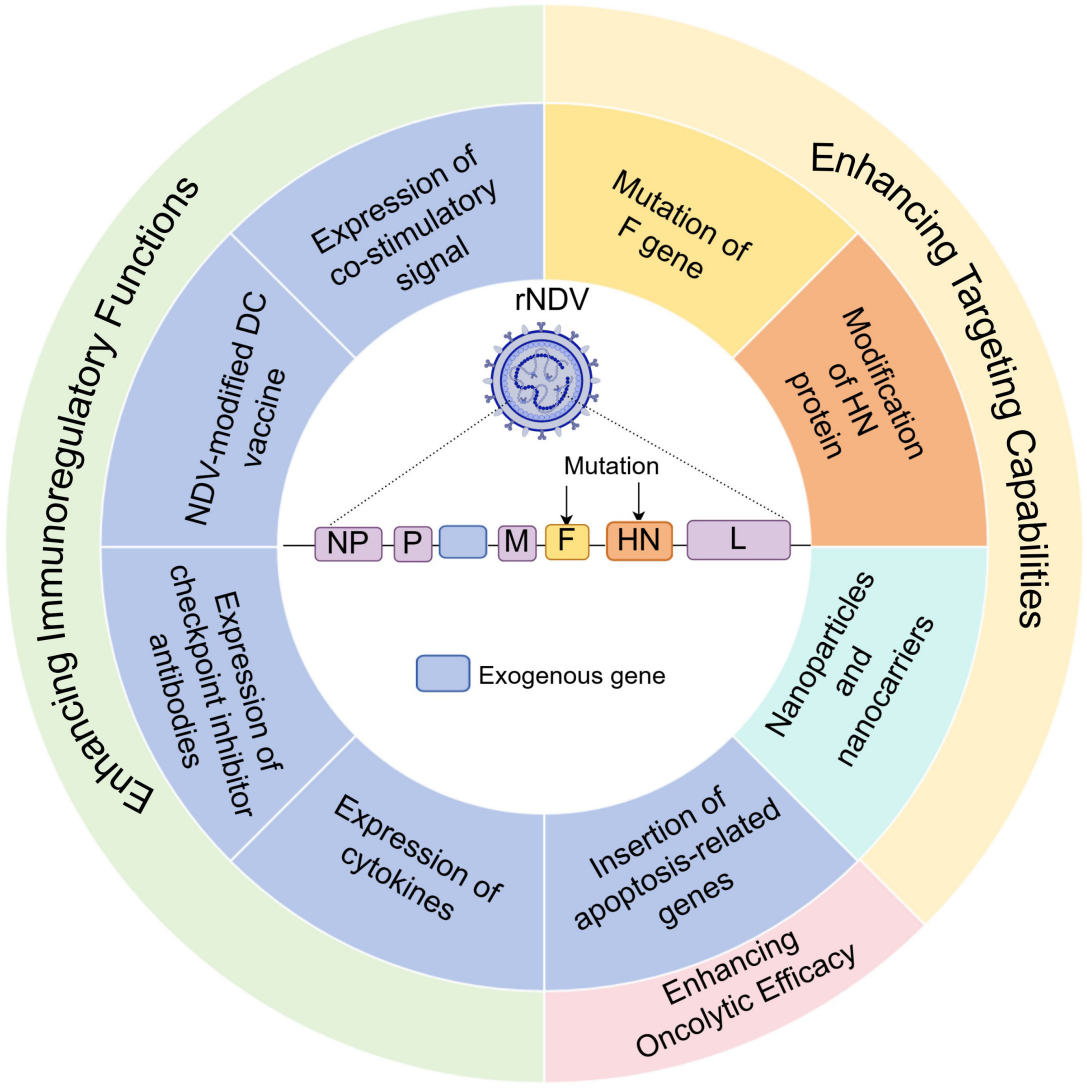
Schematic diagram of NDV modification strategies. NDV, Newcastle disease virus; rNDV, recombinant Newcastle disease virus; NP, nucleoprotein; P, phosphoprotein; M, matrix protein; F, fusion protein; HN, hemagglutinin-neuraminidase; L, large protein. Created with Figdraw.

Genetic engineering has generated a new generation of highly effective and safe recombinant NDV by removing non-essential viral genes or inserting exogenous genes. This genetic modification primarily relies on reverse genetics techniques, which involve manipulating the viral genome at the DNA level, followed by rescuing viruses with specific characteristics. Genetic modification strategies for NDV include enhancing its targeting ability by modifying viral proteins, increasing its oncolytic capacity through the insertion of apoptosis-related genes or by antagonizing antiviral pathways in tumor cells, and promoting NDV’s antitumor immune response by incorporating immunomodulatory genes (**Figure 1**, **Tables 1**, **2**).

**Table 1:** Comparison of different modifying strategies.

Strategies	Strengths	Limitations	Indications
Enhancing targeting capabilities	1. Increased tumor selectivity & safety (14) 2. Enhanced potency (16, 17)	1. Limited by target availability 2. Possibly impair viral replication or stability	Tumors with a well-defined, homogeneously expressed surface antigen.
Enhancing oncolytic efficacy	1. Direct & potent cell killing (19) 2. Synergy with immunogenic cell death (33, 47)	1. Limited immune activation 2. Safety concerns 3. Does not directly engage adaptive immunity.	Bulky, fast-growing tumors requiring rapid cytoreduction.
Enhancing immunoregulatory functions	1. Potent “*in situ* vaccination” (40) 2. Overcomes tumor immunosuppression (43, 44) 3. Induce systemic immunity (48)	1. Risk of “cytokine release syndrome” 2. Larger transgene capacity required	Immunosuppressive tumors that are refractory to current immunotherapies.

**Table 2:** Modification of NDV.

Table 2a. Enhancing targeting capabilities.							
Type of rNDV	NDV strain	Modification	Tumor type	*In vitro* experiment	*In vivo* experiment	Immune response	Reference
rVSV-NDV	Hitchner B1 strain	modified the F cleavage site (F3aa)	HCC	enhanced fusion of HCC cells *in vitro*	resulted in prolonged (35 days compared with 18 days in the control group) in HCC tumors	↑IFN-β, NK cells, myeloperoxidase positive cells	(49)
rLaSota-BC-RFP	LaSota	replaced F protein cleavage site with that from the Beaudette C strain	melanoma	reduced migration rate of B16F10 cells (52.41% ± 2.10%, compared with 66.25% ± 2.33% in the rLaSota-RFP group)	reduced tumor volume (315.42 ± 20.77 mm^3^ versus 569.71 ± 76.50 mm^3^) and improved survival rate (90% versus 70%) compared with the rLaSota-RFP group	↑ IL-12, IFN-γ, IL-15, and TNF-α, TILs	(50)
S519G rNDV	VG/GA	H domain replaced by the S519G mutant H domain	colorectal cancer	enhanced killing ability against HCT 116 cells (41.287%, compared with 29.13% in the rNDV group)	showed a stronger suppressive effect on tumor volumes (496.2 mm^3^ compared with 891.5 mm^3^ in the rNDV group)	Not done	(51)
Table 2b. Enhancing oncolytic efficacy.							
Type of rNDV	NDV strain	Modification	Tumor type	*In vitro* experiment	*In vivo* experiment	Immune response	Reference
rL-hIFN-λ1	LaSota	insertion of human IFN-λ1 gene	gastric adenocarcinoma	induced apoptosis in SGC cells	Not done	↑IFN-λ1, IFN-γ ↓IL-13	(52)
rNDV-p53	D90	insertion of human p53 gene	glioma	inhibited glioma cells *in vitro*	induced apoptosis and prolonged survival	↑cytotoxic T cells	(26)
rNDV-B1/Fas	rNDV-B1	insertion of human TNF receptor Fas gene	melanoma	induced apoptosis in B16-F10 melanoma cells	increased the rate of complete tumor remission (83% versus 12% in the rNDV-B1 group)	↑IFN-β, monocytes, neutrophils	(21)
rNDV-IL-2-TRAIL	LaSota	insertion of IL-2 and TRAIL genes	HCC; melanoma;	enhanced the apoptotic potency for B16-F10 and H22 tumor cells	reduced tumor growth (7/8 mice underwent complete regression in melanoma and HCC models compared with 1/8 in the control group)	↑INF-γ, TNF-α, CD4+ and CD8+ T cells	(33)
NDV/Anh-TRAIL	Anhinga	insertion of TRAIL gene	HCC	enhanced apoptosis (63.5% compared with 9.5% in the control group)	exhibited pronounced inhibitory efficacy *in vivo*, with an average tumor volume of 235.96 mm^3^ compared with 3261.28 mm^3^ in the control group	↑T cells	(47)
rNDV-TRAIL	VG/GA	insertion of TRAIL gene	colorectal cancer	exhibited greater apoptotic efficacy (58.56% versus 26.8%) in HT-29 cells compared with rNDV	inhibited tumor growth, with a tumor volume of 373.8 mm^3^ compared with 685.6 mm^3^ in the rNDV group	↑apoptosis	(53)
rNDV-TRAIL	LaSota	insertion of TRAIL gene	melanoma	induced apoptosis of B16-F10 cells (78.8% compared with 0.8% in the control group)	inhibited tumor growth and prolonged the survival (6/8 compared with 0/8 in the control group) of treated animals	↑apoptosis	(25)
Table 2c. Enhancing immunoregulatory functions.							
Type of rNDV	NDV strain	Modification	Tumor type	*In vitro* experiment	*In vivo* experiment	Immune response	Reference
rNDV-MIP3α	LaSota	insertion of MIP3α gene	melanoma, colon cancer	increased secretion of IFN-γ (4- to 5-fold compared with the control group) and TNF-α (fivefold compared with the control group) in CT26 and B16 cell lines	increased IFN-γ-secreting CD8+ cells (sevenfold compared with the control group)	↑IFN-γ, TNF-α, DCs, TILs	(36)
73T-R-198	73T	insertion of human GM-CSF gene	fibrosarcoma	enhanced the oncolytic activity in HT1080 fibrosarcoma cells (6-fold compared to the wild-type NDV)	led to tumor growth suppression in the HT1080 xenograft tumor model	↑ neutrophil, NK cell, and macrophage infiltration;↑IFN-β, CCL3, CCL5, IL-6, CCL2, CXCL10, IFN-γ, IL-10, TNF-α, CXCL9, and IL-12, GM-CSF	(54)
NDVmuGM-CSF	73T	insertion of murine GM-CSF gene	colon cancer	more than 40% cell death after infection	resulted in significant antitumor activity and an increase in median survival times (30.5 days versus 22 days in the control group)	↑CCL5, CXCL10, CXCL1, PD-L1, MHC-I; CD8+ T cells ↓Tregs, DCs	(55)
rNDV-IL-7	LX	insertion of IL-7 gene	melanoma	LX/(IL-7)–modified tumor cells exhibited significantly higher cytotoxicity against B16-F10 cells	increased infiltration of CD8+ and CD4+ T cells in a melanoma model	↑IL-7, IFN-γ, TILs	(56)
rNDV-IL12	AF2240-i	insertion of IL-12 gene	colon cancer	induced apoptosis of CT26 colon cancer cells *in vitro*	had smaller tumor volume (537.90 ± 12.99 mm^3^) compared with AF2240-i treated group (1113.00 ± 32.16 mm^3^)	↑IL-2, IL-12, IFN-γ, TILs	(57)
rLaSota/IL2	LaSota	insertion of human IL-12 gene	HCC; melanoma	–	inhibited tumor growth and increased the number of survivors (n = 6 in melanoma model and n = 7 in HCC model) compared with rLaSota group (n = 3 in melanoma model and n = 4 in HCC model)	↑IL-12, CD4+ and CD8+ cells; MHC II	(58)
LX/IL(15 + 7)	LX strain	insertion of IL-15 and IL-7 genes	melanoma	significantly enhanced the cytotoxicity of splenocytes	increased infiltration of CD8+ and CD4+ T cells in both prophylactic and therapeutic models	↑CD4+ T cells and CD8+ T cells	(59)
rClone30-IL24	rClone30	insertion of IL-24 gene	HCC	enhanced the oncolytic effect, apoptosis and tumor cell fusion	suppressed tumor growth, with a tumor volume increase of 95.504 mm³ compared with 674.617 mm³ in the control group	Not done	(60)
NDV-ICOSL	LaSota	insertion of murine ICOSL gene	melanoma	resulted in 40- to over 400-fold induction of ICOSL on the surface of infected cells compared to the wild-type NDV	enhanced tumor control (6/15 tumor free compared with 2/15 in the wild-type group)	↑TILs; ↓Tregs	(46)
rNDV-mOX40L	rClone30	insertion of murine co-stimulator OX40 ligand gene	colon cancer	inhibited the growth of CT26 cells by 74.43% at 10 MOI, 72 hours post-infection.	had greater inhibition rate of tumor growth (81.44% compared with 66.26% in the NDV group)	↑CD4+ and CD8+ T cells, IFN-γ	(61)
rNDV‐PTEN	–	insertion of phosphatase and tensin homolog genes	glioblastoma	induced growth inhibition and cell death of T98G glioblastoma cells	inhibited glioblastoma tumor volumes by 53%	Not done	(62)
NDV/F3aa-minigal	B1	insertion of a TAA epitope gene	colon cancer	Not done	increased the number of complete tumor regression	↑IFN-γ; CD8+ T cells	(63)
NDV-GT	LaSota	insertion of the porcine a1,3GT gene	HCC	demonstrated superior inhibitory effects	showed a high rate (90.00%) of disease control and durable responses	↑granzyme B, perforin, CD4+ and CD8+ T cells	(48)
NDV-anti-VEGFR2	rClone30	insertion of an anti-VEGFR2 gene	NSCLC	inhibited tumor cell proliferation when combined with radiotherapy	achieved a tumor growth inhibition rate of 86.48% when combined with radiotherapy	↓ HIF-1α, RAD51 and γ-H2AX	(64)
NDV-αCTLA-4; NDV-sPD-1	LaSota	insertion of the immune checkpoint inhibitor genes	Colon cancer, melanoma	oncolytic activities were detected in B16-F10 and CT26LacZ cell lines	rNDV-treated groups demonstrated 30%-50% CR and increased average survival from 13.6 to 23.9 days	↑Natural killer cells and cytotoxic T cells, ↓Tregs and MDSCs	(65)
rNDV–anti-PD1; rNDV–anti-PDL1; rNDV–anti-CD28– mIL-12; rNDV–anti-PD1–mIL-12; rNDV–anti-PDL1–mIL-12	LaSota L289A	insertion of PD-1 gene; PDL1 gene; CD28 gene; CD28 and mIL-12 genes; PD1 and mIL-12 genes; PD-L1 and mIL-12 genes	melanoma	induced cell lysis in B16-F10 murine melanoma cells	rNDV–anti-PD1, rNDV–anti-PD1–mIL-12, and rNDV–antiPDL1–mIL-12 demonstrated 37%-62% CR rates when combined with systemic anti-CTLA-4 antibody	↑IFN-α, granzyme B, TILs	(45)

IFN, interferon; IL, interleukin; TNF-α, tumor necrosis factor-α; TRAIL, tumor necrosis factor-related apoptosis-inducing ligand; HCC, hepatocellular carcinoma; MIP3α, macrophage inflammatory protein-3α; DCs, dendritic cells; TILs, tumor-infiltrating lymphocytes; GM-CSF, granulocyte-macrophage colony-stimulating factor; NK, natural killer; CCL, chemokine (C-C motif) ligand; CXCL, chemokine (C-X-C motif) ligand; PD-L1, programmed cell death ligand 1; MHC-I, major histocompatibility complex class I; Tregs, regulatory T cells; MHC II, major histocompatibility complex class II; ICOSL, inducible co-stimulator ligand; PTEN, phosphatase and tensin homolog; TAA, tumor-associated antigen; VEGFR2, vascular endothelial growth factor receptor 2; NSCLC, non–small cell lung cancer; HIF-1α, hypoxia-inducible factor-1α; γ-H2AX, γ-Histone H2AX; CTLA-4, cytotoxic T lymphocyte-associated antigen 4; CR, complete response; MDSCs, myeloid-derived suppressor cells; PD1, programmed cell death protein 1; ↑, increased number or increased level; ↓, decreased number or decreased level.

### 2.1 Enhancing targeting capabilities

Although NDV exhibits tumor tropism, engineered modifications can further restrict its infection to specific tumor cell types, enabling precise targeting. This strategy involves genetically modifying viral surface proteins to increase infection efficiency in tumor cells while minimizing infection of healthy tissues.

The fusion (F) protein plays an important role in NDV-mediated cell fusion. Research indicates that mutating the DI-DII linker region of F protein alters its fusion activity, thereby promoting cell fusion independently of the hemagglutinin-neuraminidase (HN) protein (14). This HN-independent fusion capability offers novel opportunities for targeting tumor cells with NDV.

The HN protein primarily mediates the recognition of sialic acid receptors on cell surfaces. Modifying the HN protein can further enhance NDV’s tumor-targeting capabilities. Engineering specific regions of the HN protein can enhance its binding affinity to tumor cell surface receptors, thereby increasing viral infectivity. This strategy has been successfully applied in adenoviruses (15), providing strong support for analogous modifications in NDV.

Furthermore, nanotechnologies such as nanoparticles and nanocarriers can significantly enhance NDV’s targeting and delivery efficiency (16, 17). pH-sensitive surface protonation allows NDV to remain stable at physiological pH while rapidly increasing its affinity in the acidic TME, thereby effectively targeting tumor cells (18). This surface protonation-based affinity switching mechanism is more efficient than conventional charge-switching approaches.

### 2.2 Enhancing oncolytic efficacy

The oncolytic properties of NDV primarily result from defects in the antiviral interferon (IFN) signaling pathway within tumor cells and their diminished sensitivity to signaling mediated by the type I IFN receptors (9–11). Impaired antiviral IFN signaling allows viruses to replicate efficiently, leading to direct cell lysis. Despite this, some tumors retain the ability to mount effective antiviral responses, which may partially explain their resistance to oncolytic NDV therapy. Thus, repression of IFN induction during NDV infection may allow for better viral replication in tumors, suggesting that the oncolytic activity of NDV can be enhanced by improving the virus’s ability to suppress the antiviral immune response. Research indicates that the rNDV strain, which expresses the influenza non-structural protein 1 (NS1) known for its IFN-antagonist properties, reduces IFN expression by inhibiting retinoic acid-inducible gene I (RIG-I) receptor signaling, interferon regulatory factor 3 (IRF3) dimerization, and IFN-β production (19). This enhances the virus’s ability to form syncytia and lyse tumor cells (19). Another rNDV strain expressing an IFN-antagonistic protein also demonstrated enhanced oncolytic effects compared to wild-type NDV in a mouse fibrosarcoma model (20).

NDV can trigger immunogenic cell death (ICD), which can manifest through various pathways such as necrosis, necroptosis and pyroptosis (21–24). Genetic modification of NDV-induced apoptosis can further enhance its oncolytic efficacy. When the tumor necrosis factor-related apoptosis-inducing ligand (TRAIL) gene is inserted into the NDV genome, TRAIL binds to death receptors, significantly enhancing NDV’s therapeutic effect against malignant melanoma (25). The rNDV carrying the p53 gene (rNDV-p53) exhibits significant antitumor activity both *in vitro* and *in vivo*, inducing apoptosis in glioma cells by upregulating apoptosis-related genes (26). rNDV-B1/Fas, which expresses the human tumor necrosis factor receptor Fas, enhances both endogenous and exogenous apoptotic pathways, thereby amplifying NDV’s antitumor efficacy against melanoma (21).

### 2.3 Enhancing immunoregulatory functions

CD8+ T cells play a crucial role as effector cells in the immune response against tumors. Engineered NDV expressing cytokines or immune checkpoint inhibitors (ICIs) effectively enhances local immune cell infiltration and improves antitumor immune responses. Granulocyte-macrophage colony-stimulating factor (GM-CSF), which activates antigen-presenting cells (APCs), is commonly used to modify oncolytic viruses. T-VEC, a genetically modified herpes simplex virus that expresses GM-CSF, has been approved by the FDA for the treatment of metastatic melanoma (27). The recombinant NDV strain MEDI5395 expresses human GM-CSF and has been shown to enhance the secretion of pro-inflammatory cytokines in human peripheral blood mononuclear cells (PBMCs), including IFN-α, IL-6, IL-8, and tumor necrosis factor-alpha (TNF-α) (28). Cytokines are small proteins with diverse biological activities secreted by activated immune cells. They can directly stimulate immune effector cells within the tumor microenvironment or recruit additional immune cells to the tumor site, thereby enhancing the antitumor response of cytotoxic T cells (29). Multiple rNDVs expressing IFNs or pro-inflammatory cytokines have been developed, including rNDV-IL2 (30–33), rNDV-IL24 (34), and rNDV-IFN (35). *In vitro* and *in vivo* experiments demonstrate that these rNDVs exhibit stronger antitumor effects than wild-type strains (30–35). NDV-mip3a is a rNDV expressing macrophage inflammatory protein-3α (MIP-3α). This modification enhances antitumor activity by inducing a stronger systemic immune response and modulating the TME (36). Schirrmacher et al. engineered ATV-NDV to express bispecific anti−CD28 fusion protein (bsHN-CD28) on its surface, enabling it to bind directly to CD28 on T cells and deliver co-stimulatory signals (37). In a Phase I clinical trial, fourteen patients with advanced colorectal cancer treated with bsHN-CD28-modified ATV-NDV exhibited an immunological response characterized by tumor-reactive T cells, without any serious adverse events (37).

Dendritic cells (DCs) in the TME can be activated by oncolytic viruses through multiple mechanisms, including direct viral exposure and indirect activation via products released from infected neighboring tumor cells (38). DCs serve as critical targets for initial viral detection because their pattern recognition receptors (PRRs), including RIG-I-like receptors (RLRs) and toll-like receptors (TLRs), are activated upon sensing damage-associated molecular patterns (DAMPs) and pathogen-associated molecular patterns (PAMPs). When stimulated by DAMPs and PAMPs, immature DCs upregulate the expression of major histocompatibility complex class I (MHC I) and class II (MHC II) molecules, as well as co-stimulatory molecules, leading to their maturation. These molecules facilitate the processing and presentation of antigens to naïve T cells, thereby inducing and expanding CD8+ T cell populations (39). Combining NDV with DCs enhances the antigen-presenting capacity of DCs, thereby promoting T-cell proliferation and cytokine secretion. A patient with hormone-refractory metastatic prostate cancer achieved complete remission following treatment with local hyperthermia (LHT) combined with viral oncolysate-pulsed dendritic cells (VOL-DCs) (40). This combination was shown to activate the immune system and induce a durable antitumor response. In another case report, a 70-year-old patient with invasive ductal breast cancer and liver metastases survived for more than 66 months following treatment with LHT and VOL-DCs, with no recurrence or additional metastases during this period (41).

Within the tumor microenvironment, regulatory T cells (Tregs) release inhibitory cytokines to suppress effector T cell, facilitating tumor evasion (42). Immune checkpoint inhibitors can block this effect. However, most patients do not respond to ICI therapy, primarily due to insufficient expression of target proteins in tumors or a lack of infiltrating cytotoxic T lymphocytes. On one hand, NDV induces the upregulation of immune checkpoints, including cytotoxic T-lymphocyte-associated protein 4 (CTLA-4) and programmed death-1 (PD-1). On the other hand, it triggers an inflammatory response that results in substantial infiltration of immune cells (43, 44). Consequently, combining OVs with ICIs can significantly enhance antitumor efficacy. The rNDV strains expressing checkpoint inhibitors (rNDV-anti-PD1 and rNDV-anti-PDL1) demonstrated enhanced antitumor effects when combined with CTLA-4 inhibitors, compared to the use of CTLA-4 inhibitors alone (45). NDV-ICOSL is a recombinant NDV expressing the inducible co-stimulator ligand (ICOSL), which increases the infiltration of activated T cells in both primary tumor sites and distant sites. When combined with CTLA-4 inhibitors, it significantly enhances the anti-tumor efficacy of immune checkpoint blockade therapies (46).

## 3 Anti-tumor mechanism of recombinant NDV

Recombinant NDV exhibits vastly enhanced antitumor potency. Its action transcends mere oncolysis, evolving into a multidimensional, synergistic process. The basis of rNDV’s attack on tumors is its direct oncolytic activity. This process begins with the specific binding of viral envelope proteins—such as the engineered, enhanced-targeting HN protein—to receptors on tumor cells. Subsequently, the virus enters the cell via membrane fusion or endocytosis, releasing viral ribonucleoprotein complexes (vRNPs) into the cytoplasm. The virus exploits the host cell’s transcriptional and translational machinery to synthesize substantial amounts of viral RNA and proteins, assembling them into new viral particles. Ultimately, the exocytosis and release of numerous viruses rupture the tumor cell membrane, leading to complete lysis and cell death. This process not only directly reduces tumor burden but, more importantly, induces immunogenic cell death, thereby laying the groundwork for subsequent activation of the immune system.

rNDV is capable of infecting normal cells within the tumor microenvironment, which have preserved type I IFN response. After rNDV infection, PAMPs inherent to the virus and DAMPs released by dying cancer cells are recognized by PRRs within immune cells. This activates a cascade of downstream signaling pathways involving nuclear factor kappa-B (NF-κB) and interferon regulatory factors (IRFs), leading to the rapid secretion of type I interferons and pro-inflammatory cytokines, including TNF-α, IL-6, and IL-1β (13). These cytokines recruit innate immune cells—including neutrophils, natural killer (NK) cells, and macrophages—to rapidly infiltrate the tumor. Concurrently, NK cells activated by cytokines such as IFNs directly kill tumor cells and secrete IFN-γ, further amplifying the immune response (66). High concentrations of inflammatory factors also suppress pre-existing immunosuppressive molecules within the TME, such as transforming growth factor-beta (TGF-β), and interfere with the immunosuppressive functions of Tregs and myeloid-derived suppressor cells (MDSCs). This process reprograms the TME, converting “cold” tumors into “hot” tumors and creating favorable conditions for initiating adaptive immune responses.

Inducing specific anti-tumor immunity is the primary mechanism through which rNDV exerts its long-lasting antitumor effects and prevents distant metastasis and recurrence. Upon lysis, tumor cells infected with rNDV release substantial quantities of DAMPs, such as calreticulin (CRT), ATP, and high-mobility group box 1 (HMGB1) (12). The uptake of these antigens triggers activation and maturation in cross-presenting DCs. Subsequently, activated DCs present processed antigen peptides via MHC-I and MHC-II molecules to naive CD8^+^ and CD4^+^ T cells, thereby activating and expanding large numbers of antigen-specific cytotoxic T lymphocytes (CTLs) and helper T (Th) cells. These activated T cells subsequently circulate through the bloodstream, infiltrate tumor tissues, and specifically recognize and eliminate tumor cells expressing the corresponding antigens via the perforin-granzyme pathway or the Fas/FasL pathway. The exogenous genes carried by rNDVs act as boosters in this process. GM-CSF enhances the recruitment and maturation of DCs and strengthens their antigen-presenting capabilities (28). IL-12 promotes the activation and proliferation of CTLs and NK cells (30–33). Meanwhile, immune checkpoint inhibitor single-chain variable fragments (scFv), such as anti-PD-L1, can relieve the suppression of infiltrating T cells by tumor cells (45), reversing T cell exhaustion and rendering the immune response more potent and enduring.

NDV selectively infects and lyses tumor vascular endothelial cells, playing a crucial role in tumor therapy (67). At the same time, it inhibits tumor angiogenesis by stimulating the production of TNF-α and IFN-γ (68). IFN-γ not only inhibits angiogenesis by downregulating the DLL4/Notch signaling pathway but also promotes vascular normalization through its interactions with endothelial cells, thereby enhancing antitumor immune responses (69, 70). Furthermore, TNF-α enhances immunotherapy efficacy by altering vascular endothelial barrier function and increasing T lymphocyte infiltration (71). These findings provide novel insights into NDV’s applications in tumor therapy and offer theoretical support for the development of inflammation-based antitumor strategies (72). A study demonstrates that VEGF-Trap-expressing rNDV reduces the growth rate of vascular endothelial cells by 85.37% (73). This rNDV exhibits an enhanced inhibition of colon cancer through amplified anti-angiogenesis.

**Figure 2:**
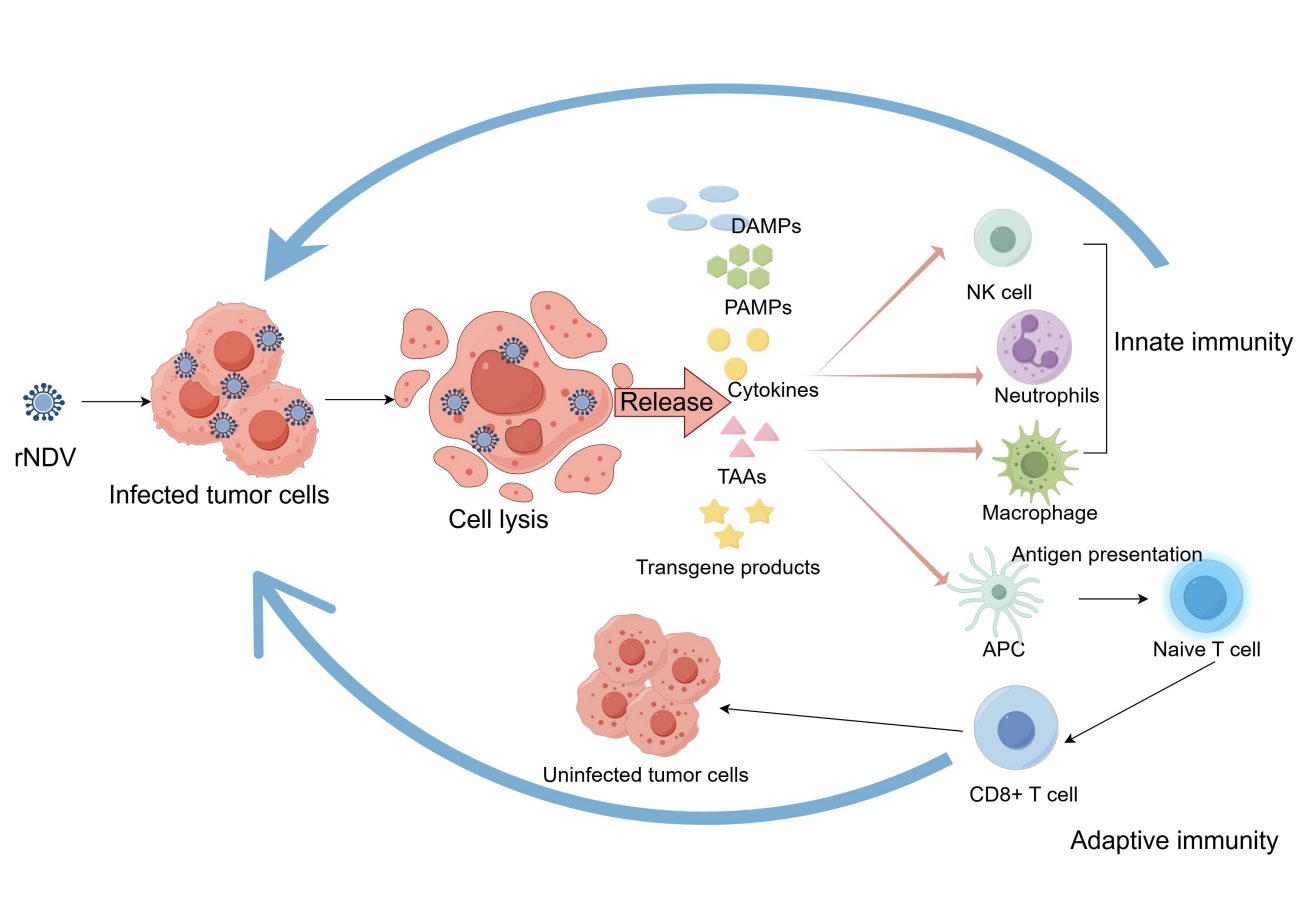
The anti-tumor mechanism of rNDV. rNDV selectively infects and lyses tumor cells. Upon lysis, tumor cells release substantial quantities of DAMPs, PAMPs and TAAs. DAMPs are recognized by immune cells, leading to the secretion of pro-inflammatory cytokines. These cytokines recruit innate immune cells, including NK cells, neutrophils, and macrophages. Meanwhile, the uptake of antigens triggers the activation and maturation of APCs. APCs present antigens to naïve T cells, thereby activating them. Ultimately, activated CD8+ T cells specifically recognize and eliminate tumor cells that express the corresponding antigens. rNDV, recombinant Newcastle disease virus; DAMPs, damage-associated molecular patterns; PAMPs, pathogen-associated molecular patterns; TAAs, tumor-associated antigens; NK, natural killer; APC, antigen-presenting cell. Created with Figdraw.

The anti-tumor mechanism of rNDV involves an interconnected, stepwise, and synergistic process. NDV not only directly lyses cells but also induces innate and adaptive immune responses in the TME (**Figure 2**). This process is further enhanced by the expression of immunoregulatory genes, ultimately resulting in potent, long-lasting anti-tumor immunity.

## 4 Applications of recombinant NDV

Recombinant NDV has demonstrated therapeutic potential in multiple cancer types, including melanoma, primary hepatocellular carcinoma, colorectal cancer, breast cancer, and glioma. Recombinant NDV LX/IL (15 + 7), which co-expresses IL-7 and IL-15, enhanced the infiltration of CD8+ and CD4+ T cells, significantly inhibiting tumor growth in melanoma (59). rNDV-MIP3α induced cellular immune responses in melanoma, as demonstrated by a sevenfold increase in IFN-γ–secreting CD8+ T cells compared to the control group (36). Recombinant NDV with a modified F cleavage site prolonged survival in primary hepatocellular carcinoma, extending it to 35 days compared to 18 days in the control group (49). rNDV-IL12 suppressed the growth of colon cancer, resulting in a smaller tumor volume (537.90 ± 12.99 mm³) compared to that of the AF2240-i-treated group (1113.00 ± 32.16 mm³) (57). rNDV-IL12 modulated immune responses by increasing the levels of CD4+ T cells, CD8+ T cells, IL-2, IL-12, and IFN-γ (57). Recombinant NDV expressing IL-12 (rAF-IL12) resulted in 52% inhibition of breast cancer growth, whereas AF2240 caused 34.5% inhibition (74). Recombinant NDV expressing the human p53 gene has been shown to induce apoptosis in glioma cells, enhance cytotoxic T cell infiltration, and prolong survival in glioma-bearing mice (26). These studies provide evidence for the further application of rNDV in solid tumor therapy.

Clinical trials of NDV have demonstrated its efficacy and safety (**Table 3**). MEDI5395, a recombinant NDV expressing the human GM-CSF gene, demonstrated antitumor activity in Phase I clinical trials. When combined with durvalumab, it exhibited favorable safety and efficacy profiles in patients with advanced-stage tumors (75). NDV-GT, a recombinant NDV expressing porcine α1,3GT gene, induced surface expression of the αGal antigen on tumor cells. Pre-existing anti-αGal antibodies in the human body rapidly recognized this antigen, triggering a hyperacute immune rejection response that causes tumor vascular embolism and extensive tumor cell death. Results from a Phase I clinical trial reported in 2025 indicated that 90% of advanced cancer patients treated with NDV-GT achieved disease control without experiencing severe adverse effects (48). The clinical safety of NDV has been extensively demonstrated by data from multiple trials. No serious adverse events were reported in the clinical trials of NDV therapies. While current preclinical studies and preliminary clinical outcomes are encouraging, most rNDV therapies remain in preclinical or early clinical stages. Their safety, efficacy, and optimal application strategies require validation through further large-scale clinical trials.

**Table 3:** Clinical trials of NDV.

NDV strain	Route of administration	Dosage	Type of cancer	Phase	Safety	Clinical outcome	References
73T	Subcutaneous injection	Allogenic or autologous human melanoma cells infected with NDV	melanoma	II	No adverse effects were reported.	10 years of survival was above 60% compared with 6-33% in historic control.	(76, 77)
MTH-68	Inhalation	4*10^3^PFU	Advanced cancers	II	–	1-year survival for 22 out of 33 versus 4 out of 26 in control group	(78)
HUJ	Intravenous administration	Dose escalation up to 5.5*10^10^EID_50_	Glioblastoma multiforme	I	Five patients developed fever, and eight patients had neurological seizures.	1/11 achieved a complete response, others had progressive disease	(79)
PV 701	Intravenous administration	Dose escalation up to 1.20*10^11^PFU	Advanced cancers	I	Tumor site-specific adverse events and acute dosing reactions were observed but not cumulative toxicity.	Objective responses occurred at higher dosage; progression-free survival ranged from 4 to 31 months	(80)
PV 701	Intravenous administration	Dose escalation up to 1.20*10^11^PFU	Advanced cancers	I	Flu-like symptoms were observed in all patients. But no dose-limiting toxicities were observed.	1/16 experienced near-complete response	(81)
PV 701	Intravenous administration	1.20*10^11^PFU	Advanced cancers	I	Three side effects were seen: flu-like symptoms; tumor-site-specific adverse events; and infusion reactions.	6 out of 18 responses	(82, 83)
ATV-NDV	Intracutaneous vaccination	1*10^7^ ATV-NDV cells	Head and neck squamous cell carcinoma	III	Flu-like symptoms, mild fatigue and palpable indurations at the vaccination site were reported.	Percentages of survival of vaccinated patients with stage III and stage IV tumors (n = 18) were 61% at 5 years.	(84)
ATV-NDV	Intracutaneous vaccination	1*10^7^ ATV-NDV cells	Glioblastoma	III	Mild fever, temporary cholestasis, and fatigue.	The median PFS was 40 weeks, and the median OS was 100 weeks.	(85)
ATV-NDVαHN-αCD28	Intracutaneous vaccination	Dose escalation up to 1 μg of bsHN-CD28 protein added to the ten million ATV-NDV vaccine cells	Stage IV colorectal cancer	I	No severe adverse events were recorded.	The decrease in CEA and the partial response of metastases in four patients were observed.	(37)
MEDI5395 (rNDV-GM-CSF)	Intravenous administration	Dose escalation up to 1*10^11^ PFU	Advanced solid tumors	I	All 39 patients experienced ≥1 TEAE, most commonly fatigue (61.5%), nausea (53.8%) and chills (51.3%). Grade 3–4 TEAEs occurred in 27 (69.2%) patients.	Four patients (10.3%) achieved a partial response.	(75)
MEDI9253 (rNDV-IL12)	–	–	Solid tumors	I	Study results have not been published.	Study results have not been published.	NCT04613492
NDV-GT	Intravenous administration	Dose escalation up to 1*10^9^ PFU/kg	Relapsed metastatic cancers	–	No serious adverse events were reported.	NDV-GT demonstrated a high disease control rate of 90.00% and durable responses in 20 patients.	(48)

PFU, plaque forming unit; EID50, 50% egg infectious dose; ATV-NDV: the NDV-modified autologous tumor vaccine; PFS, progression-free survival; OS, overall survival; ATV-NDV-αHN-αCD28: the ATV-NDV strain expressing the anti-CD28 fusion protein, coupled to viral HN anchor molecules; CEA, carcinoembryonic antigen; TEAEs, treatment-emergent adverse events; NDV-GT, a recombinant NDV strain with porcine a1,3GT gene.

## 5 Combination strategies

Due to tumor heterogeneity, mutation burden, and variations in the TME, monotherapy-based tumor immunotherapy often leads to resistance, limiting the ability of OVs to achieve optimal antitumor efficacy. Combining OV therapy with other treatment modalities—such as chemotherapy, ICIs, adoptive cell therapy (ACT), and radiotherapy—may be essential for improving therapeutic outcomes.

The strategy of combining oncolytic viruses with chemotherapy remains a topic of controversy. Some clinical studies suggest that the combination of oncolytic viruses and chemotherapy can achieve satisfactory antitumor effects (86–90), but a large-scale clinical trial has produced conflicting results (91). Eigl et al. reported that metastatic prostate cancer patients treated with the oncolytic virus Pelareorep plus docetaxel had a median survival of 19.1 months, compared to 21.1 months for patients receiving docetaxel monotherapy (91). Another clinical trial found that Pelareorep combined with FOLFOX/Bevacizumab resulted in worse progression-free survival (PFS) compared to monotherapy (92). These conflicting results do not demonstrate the superiority of combining oncolytic viruses with chemotherapy over chemotherapy alone, suggesting that the combination may exhibit a “1 + 1 < 1” effect.

**Figure 3:**
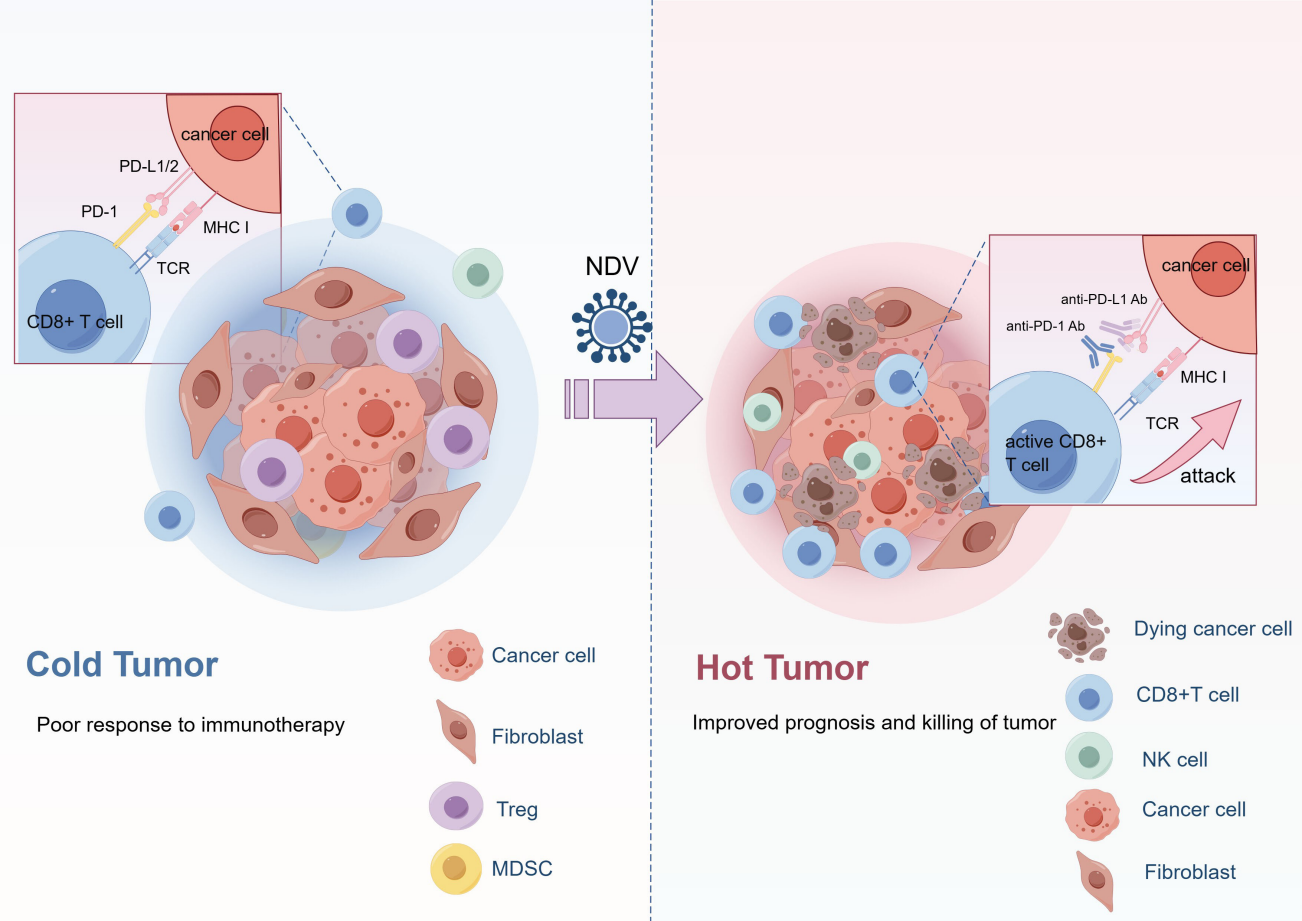
The mechanism of combination therapy. The combination of oncolytic NDV and immune checkpoint inhibitors synergistically promotes the anti-tumor effect. NDV promotes the infiltration of CD8+ T cells and natural killer cells and upregulates the expression of PD-1 and PD-L1. Meanwhile, PD-1/PD-L1 inhibitors block the PD-1/PD-L1 pathway, thereby preventing tumor cells from evading the immune response. PD-1, programmed death-1; PD-L1/2, programmed death-ligand 1/2; TCR, T cell receptor; MHC I, major histocompatibility complex class I; NDV, Newcastle disease virus; Ab, antibody; NK, natural killer; Treg, regulatory T cell; MDSC, myeloid-derived suppressor cell. Created by Figdraw.

The combination of NDV with immunotherapy currently represents the most promising strategy. Possible reasons for the limited effectiveness of ICIs include a relatively low mutation burden, minimal T-cell infiltration, and an immunosuppressive TME. Studies have demonstrated that following NDV treatment, immune checkpoints such as CTLA-4 and PD-1 are significantly upregulated on infiltrating T cells in both the injected tumors and distant tumors (43, 44). Furthermore, the expression of programmed cell death ligand 1 (PD-L1) is increased in tumor cells, myeloid cells, and stromal cells following NDV infection (44). These findings suggest that combining NDV with ICIs may enhance antitumor efficacy (**Figure 3**). Co-administration of NDV with PD-1 or PD-L1 antibodies significantly improves the therapeutic response in melanoma, resulting in 90–100% tumor-free rates compared to 70% in the NDV monotherapy group (44). Another study demonstrates the superior efficacy of NDV combined with CTLA-4 antibodies compared to monotherapy in treating melanoma, colorectal cancer, and prostate cancer (43). Recombinant NDV expressing inducible co-stimulator ligand (NDV-ICOSL) increases infiltration of GrB+ICOS+CD8+ T cells. When combined with CTLA-4 antibodies, it demonstrated a 74% tumor-free rate in distant tumors, which was superior to the combination of wild-type NDV with anti-CTLA-4 (46). Although the combination strategy of rNDV and ICIs has shown promising results in preclinical studies, further prospective clinical trials are required to validate the efficacy.

ACT has demonstrated significant efficacy in treating leukemia, melanoma, and lymphoma. However, its application to epithelial tumors is limited due to challenges related to cell infiltration and tumor heterogeneity (93). Combining OVs with ACT could potentially modify the TME of solid tumors, thereby overcoming current limitations. Chen et al. compared the efficacy of OV monotherapy with combination therapies involving chimeric antigen receptor T (CAR-T) cells and innate-like natural killer T (iNKT) cells. The results demonstrated the superiority of combination therapy over monotherapy (94). An oncolytic virus expressing truncated CD19 (CD19t) enhanced CAR-T cell targeting of solid tumors and further stimulated local antitumor immunity (95). On the other hand, CAR-T cells can serve as vectors for oncolytic viruses, delivering these viruses to tumor cells that express specific antigens. Delivering the myxoma virus to tumor cells expressing homologous antigens via CAR-T cells induces targeted tumor cell death and autophagy, resulting in tumor regression (96). These findings suggest that oncolytic viruses could be powerful tools to overcome limitations in adoptive cell therapy.

Other combination strategies include pairing with radiotherapy and targeted therapy. Results from a Phase I clinical trial of OPB301 combined with radiotherapy in esophageal cancer patients demonstrated an objective response rate (ORR) of 91.7% in 13 patients, with a favorable safety profile (97). The Phase III clinical trial of JX-594 combined with sorafenib for advanced hepatocellular carcinoma (NCT02562755) reported an ORR of only 19.2%, which was lower than the 20.9% observed in the sorafenib monotherapy group (98). Despite promising preclinical data suggesting that JX-594 could sensitize tumors to subsequent therapy with vascular endothelial growth factor (VEGF) inhibitors or vascular endothelial growth factor receptor (VEGFR) inhibitors, the trial results indicated that the combination did not demonstrate greater clinical benefit compared to sorafenib monotherapy (98, 99). Therefore, additional prospective clinical trials are necessary to validate the efficacy of NDV in combination with radiotherapy or targeted therapies.

## 6 Challenges and prospects

Compared to other oncolytic viruses, NDV has unique features: (1) NDV is a single-stranded negative-sense RNA virus that replicates in the cytoplasm, independently of the host cell's DNA replication machinery. It cannot integrate with the host genome. (2) While NDV replicates efficiently within tumors, it does not replicate in the normal cells of mammalian hosts. (3) NDV is cost-effective to produce, can be administered through various methods, and has minimal side effects.

NDV has demonstrated promising antitumor properties and a high safety profile in both preclinical and clinical studies. Despite its promising potential, studies of NDV therapy face significant challenges related to heterogeneity and methodological inconsistency, which complicate interpretation and hinder progress in the field. The primary issue is the profound heterogeneity of viral platforms and study designs across different investigations. Researchers have employed diverse reverse genetics systems, parental viral strains, and transgene payloads, which offer technical advantages during exploratory phases. However, this diversity has become a critical drawback—variations in viral backbones, production methods, and genetic modifications, making meaningful comparisons across trials nearly impossible. Moreover, early clinical trials frequently suffer from small sample sizes, single-arm designs, and a lack of standardized efficacy endpoints. Consequently, observed clinical outcomes are susceptible to confounding factors, preventing clear attribution to the oncolytic NDV itself. A third critical issue is the absence of reliable biomarker data to validate the NDV’s mechanism of action in patients. In most trials, it remains unclear whether limited efficacy results from the virus failing to infect and replicate within tumors, premature clearance by the host immune system, or an inability to overcome the immunosuppressive TME. The inconsistencies and limitations discussed above highlight the most urgent challenges currently facing the NDV field. Overcoming these hurdles is essential to translating preclinical promise into clinical reality. Therefore, future research must strategically focus on addressing the following key issues:

1. NDV is a persistent threat to the poultry industry.
2. The delivery of NDV should be optimized.
3. Complement and neutralizing antibodies compromise the therapeutic efficacy.
4. The side effects associated with NDV continue to warrant attention.
5. How to overcome the tumor heterogeneity is a problem that needs to be addressed.
6. Searching for biomarkers that can predict NDV’s efficacy is crucial.

Although NDV is of low pathogenicity in humans, it is highly contagious among poultry, which is a persistent threat to the poultry industry. The manufacture and use of NDV may result in its release into the environment, thereby necessitating regulatory oversight. Evaluating an oncolytic NDV for environmental safety is also important. However, there are few studies on the environmental shedding of NDV. Further research should be conducted and thoroughly documented.

Current delivery approaches include local delivery and systemic delivery. Most oncolytic virotherapies are administered through intratumoral injection. However, this method has specific drawbacks, including (1) Intratumoral injection may cause bleeding and unwanted metastasis at the lesion site. (2) Difficulties in accessing deep tumor tissues significantly limit the number of applicable cases. Thus, intravenous administration is essential for treating deep-seated or metastatic tumors. However, following intravenous injection, viral particles encounter multiple barriers. First, complement proteins and neutralizing antibodies in the bloodstream can neutralize and eliminate NDV before it reaches the tumor site. Second, the elevated pressure within the tumor stroma and the dense extracellular matrix hinders effective viral penetration and diffusion. Consequently, only a small fraction of the virus successfully reaches and infiltrates the tumor, thereby limiting its therapeutic efficacy. Altering the capsid, applying polymer coatings (100), or enhancing the extracellular envelope of NDVs can improve their shielding (101). Another effective strategy for intravenous injection involves encapsulating NDVs within cellular vectors or loading them onto these cells. Mesenchymal stem cells (MSCs) are promising candidates for delivering oncolytic viruses because they can transport viral particles, serve as factories for viral replication, and modulate the immune system (102–106).

NDV itself, as a foreign antigen, rapidly induces a strong antiviral immune response. Complement and neutralizing antibodies in the bloodstream can neutralize and eliminate NDV before it reaches the tumor site, severely compromising therapeutic efficacy. This may render subsequent doses ineffective, hindering the implementation of repeated, multi-course intensive therapy. Overcoming or circumventing host innate immunity represents the primary challenge in enhancing NDV efficacy. Employing cellular vector systems for NDV delivery represents a highly promising strategy. Cells such as mesenchymal stem cells (MSCs), cytokine-induced killer cells (CIK), or CD8+ T cells, which exhibit natural chemotaxis towards tumors, can serve as viral carriers. These cells carry and protect the virus, effectively evading neutralizing antibodies and complement attacks until reaching the tumor, where they release progeny viruses, achieving tumor-targeted delivery and efficient infection.

Regarding safety, the most frequently reported side effects in OV clinical trials include fever, fatigue, nausea, flu-like symptoms, and pain at the injection site. There is an overall incidence of adverse events of 26.6% associated with OV therapy (107). Compared to other immunotherapy products, OV has a more favorable safety profile. However, due to the heterogeneity both between and within tumors, the absence of specific receptors or promoters in tumor cells may lead to off-target effects, resulting in immune-related adverse events (108). The risks increase significantly when NDV is used in combination with immune checkpoint inhibitors or is modified to express immune modulators (109, 110). Cytokine release syndrome (CRS), a serious adverse effect, can also be triggered by OV therapy (111–113). Essential strategies to minimize side effects include enhancing tumor targeting through genetic engineering, dosing guided by pharmacokinetic principles, intratumoral administration to reduce systemic toxicity, and a low-dose fractionated schedule optimized for efficacy (114–116).

Cancer cells exhibit high heterogeneity in receptor expression, integrity of interferon pathways, and proliferative states. One the one hand, NDV infection requires attachment to cell surface receptors, primarily sialic acid-containing glycoproteins. There is considerable variability in the expression levels of these receptors both between different types of cancer and among patients with the same type of cancer. The absence or low expression of receptors in tumors renders them resistant to infection, posing a significant barrier to oncolysis. On the other hand, most cancer cells have defects in their IFN signaling pathways, which allows NDVs to replicate efficiently within them. However, the impairment is typically not absolute. Cancer cells with an intact IFN pathway can mount an effective antiviral response, clearing the virus and resulting in treatment resistance. In addition, the highly immunosuppressed TME lacks T-cell infiltration. Even when NDV lyses tumor cells and releases antigens, it fails to effectively initiate specific anti-tumor immune responses, thus limiting therapeutic efficacy. Hence, developing strategies that combine therapies for synergistic effects is a major research objective. Chemotherapy and radiotherapy can induce ICD, further promoting tumor antigen release and complementing NDV’s immune-activating effects. Combination with immune checkpoint inhibitors can relieve T-cell suppression, facilitating the successful removal of PD-L1-positive tumor cell subpopulations. This synergy addresses tumor heterogeneity.

Biomarkers play a crucial role in advancing individualized precision oncology by enabling clinicians to provide care that is more effective, less toxic, and more cost-efficient. However, research on biomarkers for oncolytic viruses remains limited. It is necessary to search for biomarkers that can predict the efficacy of NDV, such as pre-existing antibody titers, tumor viral receptor expression levels, IFN signaling pathway status, immune cell infiltration, post-treatment viral replication in peripheral blood, and cytokine dynamics. This will enable the identification of populations likely to benefit, facilitate real-time efficacy monitoring, and allow timely adjustment of treatment strategies.

In summary, this review has outlined the modification strategies, antitumor mechanisms, clinical translation, combination strategies, and challenges of rNDV immunotherapy. **Table 4** provides a quick reference summary of all that this review entails.

**Table 4:** Advancements in recombinant NDV for cancer therapy.

Topics	Key points	Relevant Section/Figure
Modification strategies of NDV	1. Enhancing targeting capabilities 2. Enhancing oncolytic efficacy 3. Enhancing immunoregulatory functions	Section 2
Anti-tumor mechanisms of rNDV	1. Direct oncolysis 2. Activates the innate and adaptive immune responses (65) 3. Inhibits tumor angiogenesis (67)	Section 3; **Figure 2**
Immune effects	1. Promotes the secretion of pro-inflammatory cytokines (54) 2. Increases the infiltration of CD4+ T cells, CD8+ T cells, and NK cells (48, 58, 61) 3. Decreases the infiltration of Tregs and MDSCs (65)	Section 3
Preclinical and clinical outcomes	1. Inhibits tumor growth 2. Prolongs survival time	Section 4
Challenges	1. Environmental safety 2. Routes of administration 3. Complement and neutralizing antibodies 4. Immune-related adverse effects 5. Tumor heterogeneity 6. Predictive biomarkers	Section 6

## 7 Conclusion

In conclusion, advancements in genetic engineering technology have enabled the enhancement of NDV’s targeting potential through the modification of viral proteins, as well as the improvement of its immune-stimulating capabilities by inserting exogenous genes. However, numerous unresolved challenges remain in NDV-based cancer therapy, including determining the most effective delivery route, developing strategies to evade neutralizing antibodies, overcoming tumor heterogeneity, and identifying relevant biomarkers. Future research priorities will focus on four key areas: the rational design of a new generation of rNDVs armed with potent immunomodulatory transgenes; the use of cellular vehicles for targeted delivery; the development of bispecific NDVs capable of redirecting immune effector cells; and the creation of synergistic therapeutic regimens that combine NDV with immune checkpoint inhibitors.
